# An Analytic Model for Negative Obstacle Detection with Lidar and Numerical Validation Using Physics-Based Simulation

**DOI:** 10.3390/s21093211

**Published:** 2021-05-05

**Authors:** Christopher Goodin, Justin Carrillo, J. Gabriel Monroe, Daniel W. Carruth, Christopher R. Hudson

**Affiliations:** 1 Center for Advanced Vehicular Systems, Mississippi State University, Mississippi State, MS 39762, USA; dwc2@cavs.msstate.edu (D.W.C.); chudson@cavs.msstate.edu (C.R.H.); 2 Geotechnical and Structures Laboratory, US Army Engineer Research and Development Center, Vicksburg, MS 39180, USA; Justin.T.Carrillo@erdc.dren.mil (J.C.); John.G.Monroe@erdc.dren.mil (J.G.M.)

**Keywords:** lidar, autonomy, navigation, UAV

## Abstract

Negative obstacles have long been a challenging aspect of autonomous navigation for ground vehicles. However, as terrestrial lidar sensors have become lighter and less costly, they have increasingly been deployed on small, low-flying UAV, affording an opportunity to use these sensors to aid in autonomous navigation. In this work, we develop an analytical model for predicting the ability of UAV or UGV mounted lidar sensors to detect negative obstacles. This analytical model improves upon past work in this area because it takes the sensor rotation rate and vehicle speed into account, as well as being valid for both large and small view angles. This analytical model is used to predict the influence of velocity on detection range for a negative obstacle and determine a limiting speed when accounting for vehicle stopping distance. Finally, the analytical model is validated with a physics-based simulator in realistic terrain. The results indicate that the analytical model is valid for altitudes above 10 m and show that there are drastic improvements in negative obstacle detection when using a UAV-mounted lidar. It is shown that negative obstacle detection ranges for various UAV-mounted lidar are 60–110 m, depending on the speed of the UAV and the type of lidar used. In contrast, detection ranges for UGV mounted lidar are found to be less than 10 m.

## 1. Introduction

Negative obstacle detection has been a challenge for autonomously navigating (self-driving) off-road unmanned ground vehicles (UGV) for several decades [1,2,3,4]. The difficulty associated with detecting negative obstacles is primarily geometric. Early analysis showed that the angle subtended by a positive obstacle at a range *R* from the sensor ∝1/R, whereas the angle subtended by a negative obstacle ∝$1/R^{2}$ [3]. A key development in UGV technology in the last decade has been the availability of low-cost, high resolution 3D lidar systems [5]. These systems enable precise measurements of scene geometry that spurred the development of new techniques for negative obstacle detection [6,7]. Concurrently, the development of low-cost multi-rotor unmanned aerial vehicles (UAV) has enabled multi-agent collaborative navigation using both UAV and UGV systems for sensing and mapping the terrain [8,9].

In this work, a high-fidelity, physics-based simulation for lidar sensors [10] is used to demonstrate the improvement in negative obstacle detection that can be made by incorporating lidar data acquired from a low-flying UAV into autonomous UGV navigation. In addition, a detailed analytical model for negative obstacle detection is developed. This model improves upon past methods by implementing equations that are valid for all angles (not just small angles) and taking into account the influence of vehicle speed and sensor rotation. The model is cross-validated with physics-based lidar simulation using the MSU Autonomous Vehicle Simulator (MAVS) [10].

## 2. Related Work

There has been significant work on negative obstacle detection with lidar in the last two decades. Dellenback et al. [11] showed how the elevated view angle from a UAV improves negative obstacle detection in UAV-UGV cooperative teams. Later, Shang et al. [12] showed how the inclusion of multiple lidar sensors mounted at different angles could lead to better characterization of the negative obstacles. Kim et al. [13] demonstrated that multiple UAV could be used to map terrain geometry with stereo vision. Novel techniques have also been developed to estimate occlusion effects with ground-based lidar [14].

In addition, simulation capabilities for lidar have advanced in recent years, with Yun et al. [15] using lidar simulation to optimize and virtualize scanning patterns for lidar in the detection of total leaf area in tree crowns. Furthermore, Shan et al. [16] have recently proposed a simulation-based method for achieving “super-resolution” by combining multiple virtual lidar sensor feeds.

More recently, Nakano et al. [9], Ravi et al. [17] and Péntek et al. [18] have performed fundamental analysis of lidar performance for various detection tasks, while Roberts et al. [19], Azevedo et al. [20] and Gilhuly and Smith [21] investigated the capability of UAV-mounted lidar in classifying ground points, detecting power lines, and terrain mapping, respectively. Additionally, Kandath et al. [22] have shown the viability of incorporating sensor information from a low-flying UAV into UGV path planning algorithms.

## 3. Analytical Model for Predicting Negative Obstacle Detection

Past work [3,6] has used geometric analysis to aid in the development of negative obstacle detection algorithms and investigate their limitations. In this work, we extend this past research to develop a predictive model for negative obstacle detection with lidar. This predictive model can be used to assess the optimal deployment of UGV-mounted or UAV-mounted lidar for negative obstacle detection. Table 1 summarizes the symbols used in the following analysis, while Table 2 shows the sensor properties considered in the model.

**Figure 1:**
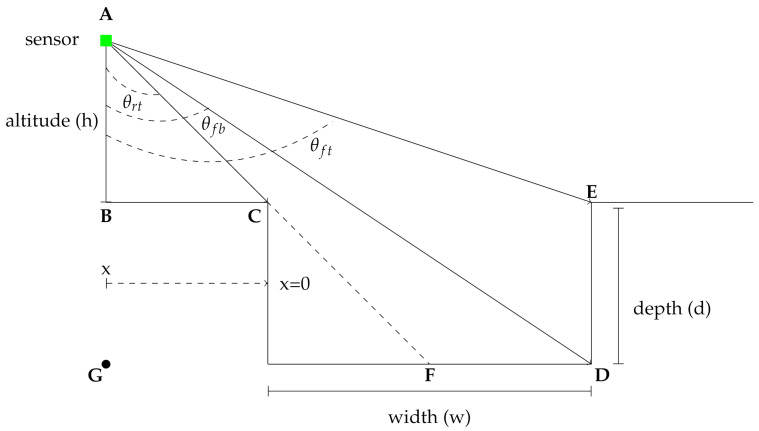
Side view of the ground coverage metric calculation. Points **A**, **B**, **C**, **D**, **E**, **F**, and **G** are defined to aid the reader in following the derivations of Equations (1)–(3).

The geometry of the model is shown in the schematic in Figure 1. Note that mount angle γ is defined with respect to nadir such that γ=0 is downward-looking, γ=π/2 is forward looking, and γ=−π/2 is rearward looking. The sensor, indicated by the green square, is mounted at an altitude of *h* meters above the ground. Note that this analysis is valid for all ranges of *h*, so the results can be applied both to UAV- and UGV-mounted sensors.

In the following analysis, equations for the number of points on the wall and floor/ bottom of a rectangular negative obstacle are developed. While prior models have primarily focused on UGV mounted sensors where h<<R, in which case the small angle approximation can be used to simplify the equations, the following analysis does not make this approximation and can therefore be used for both UAV- and UGV-mounted sensors. For UGV navigation, the primary factor of interest is the range at which the negative obstacle can be detected, which in turn places a limit on the speed of the vehicle based on the stopping distance [3]. Therefore, this analysis focuses on the detection range of the obstacle as the primary metric for performance.

For a sensor located at the horizontal coordinate *x* (where *x* is negative in Figure 1) and vertical coordinate *h*, from triangle **ABC**, the angle between the sensor and the nearest point on the negative obstacle, $\theta_{rt}$, is given by

(1) $$\theta_{rt}\left(x\right)=\tan^{-1}\left(-x/h\right)$$

In addition, from triangle **ABE**, the angle between the sensor and the farthest point on the obstacle, $\theta_{ft}$, is given by

(2) $$\theta_{ft}\left(x\right)=\tan^{-1}\left(\left(w-x\right)/h\right)$$

The angle from the sensor to the farthest point on the bottom of the obstacle, $\theta_{fb}$, is found from triangle **AGD**. However, the range must be restricted because point **D** will not be visible when the sensor horizontal position exceeds *w* (the sensor is past the hole) or is farther away than −hw/d (the front edge of the hole, **C**, obscures **D**). Therefore

(3) $$\theta_{fb}\left(x\right)=\left\{\begin{array}{cc} \theta_{rt}\left(x\right), & x\leq -hw/d,\Delta \mathrm{AGF}\\ \tan^{-1}\left(w-x\right)/\left(h+d\right), & -hw/d<x\leq w,\Delta \mathrm{AGD}\\ \theta_{ft}\left(x\right), & x>w,\Delta \mathrm{ABE} \end{array}\right.$$

**Figure 2:**
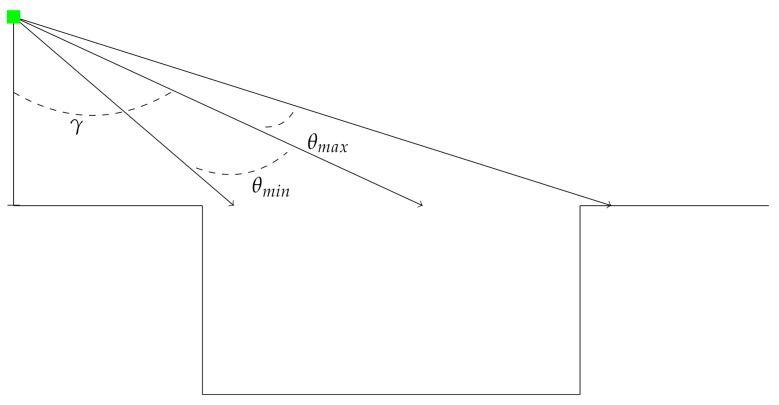
Depiction of the sensor mount angle, γ and the sensor limit function.

Automotive lidar sensors typically have a fairly narrow vertical field-of-view, so the view-angle calculations are constrained by the sensor field of view. Therefore, the sensor limit function (Figure 2) is defined as

(4) $$\theta_{lim}\left(\theta \right)=\min\left(\max\left(\theta ,\gamma +\theta_{min}\right),\gamma +\theta_{max}\right)$$

Lidar sensors also have a finite range. In order to account for the limits of the sensor range *R*, the following calculation is only valid on the range $x=[x_{0},x_{1}]$, where

(5) $$x_{0}=-\sqrt{R^{2}-{\left(h+d\right)}^{2}}$$

and

(6) $$x_{1}=w+\sqrt{R^{2}-{\left(h+d\right)}^{2}}$$

For a single scan from a location *x*, the number of points on the far wall, $n_{f}^{\prime }$ is given by

(7) $$n_{f(2D)}^{\prime }\left(x\right)=\left(\theta_{ft}\left(x\right)-\theta_{fb}\left(x\right)\right)/\delta_{v}$$

The number of points on the bottom of the negative obstacle, $n_{b}^{\prime }$, is given by

(8) $$n_{b(2D)}^{\prime }\left(x\right)=\left(\theta_{fb}\left(x\right)-\theta_{rb}\left(x\right)\right)/\delta_{v}$$

where $\delta_{v}$ is the vertical angular resolution of the sensor.

While the previous calculations were done for the two dimensional case as shown in Figure 1, it is straightforward to extend the calculations to 3D.

**Figure 3:**
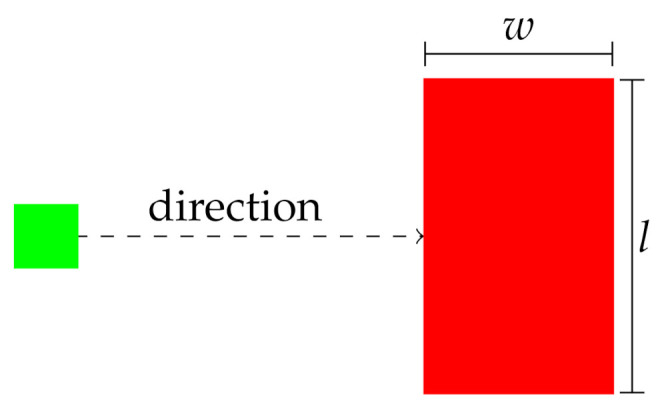
Top-down view of the hole dimensions. The green square represents the sensor, the red square represents the hole.

If the horizontal resolution of the sensor is $\delta_{h}$ and the length of the hole perpendicular to the path of travel is *l* (as depicted in Figure 3), then the horizontal angle subtended by the sensor on the hole is

(9) $$\theta_{h}=2\tan^{-1}\left(l/2x\right)$$

The total number of points in two dimensions is then scaled by a factor of $\frac{\theta_{h}}{\delta_{h}}$. With this scaling, the number of points on the front wall in three dimensions is then

(10) $$n_{f}^{\prime }\left(x\right)=\theta_{h}\left(\theta_{ft}\left(x\right)-\theta_{fb}\left(x\right)\right)/\left(\delta_{v}\delta_{h}\right)$$

The number of points on the bottom of the negative obstacle in three dimensions, $n_{b}^{\prime }$, is given by

(11) $$n_{b}^{\prime }\left(x\right)=\theta_{h}\left(\theta_{fb}\left(x\right)-\theta_{rt}\left(x\right)\right)/\left(\delta_{v}\delta_{h}\right)$$

The variables $n_{f}^{\prime }$ and $n_{b}^{\prime }$ will be referred to as the instantaneous front and bottom coverage indexes in the remainder of this work.

While the single scan totals are interesting, in most situations successive scans will be merged into a point cloud. In this case, the number of points accumulated on the obstacle as the vehicle moves toward it is of greater interest. Lower speeds will result in more accumulated points as the sensor approaches the negative obstacle, while higher speeds will result in fewer accumulated points. The number of scans for a sensor with a scan rate of $f_{s}$ moving at a speed of *v* is

(12) $$n_{s}\left(x\right)=\left\lceil \frac{\left(x-x_{0}\right)f_{s}}{v}\right\rceil$$

With this, the cumulative number of points as the sensor approaches the negative obstacle is then given by

(13) $$n_{f}\left(x\right)=\sum_{n=0}^{n_{s}\left(x\right)}n_{f}^{\prime }\left(x_{0}+nv/f_{s}\right)$$

for the far wall. For the bottom of the hole, the accumulated number of points is given by

(14) $$n_{b}\left(x\right)=\sum_{n=0}^{n_{s}\left(x\right)}n_{b}^{\prime }\left(x_{0}+nv/f_{s}\right)$$

These will be referred to as the cumulative front and bottom coverage indexes in the remainder of this work. In the following section, it will be shown that the performance of a negative obstacle detection algorithm is related to these indexes.

## 4. Negative Obstacle Detection Method

Negative obstacles can be detected by calculating the curvature of the terrain surface on a regular 2D grid. Regions on the grid that have a curvature that exceeds a certain threshold are flagged as obstacles. Obstacle regions with a height below the local average are flagged as negative obstacles.

After each scan, points are registered to world coordinates using the current odometry from the real-time kinematic localization sensor [23,24]. Each point is placed in a cell, and only the lowest points are saved in the 2D array of surface heights, z(x,y). The curvature at cell (i,j) is calculated using a five point stencil according the equation.

(15) $$\kappa_{ij}=\frac{z(i+1,j)+z(i-1,j)+z(i,j+1)+z(i,j-1)-4z(i,j)}{\Delta^{2}}$$

where Δ is the resolution of the grid. The resolution of the grid is chosen as an input parameter to the model. For AGV navigation, the most relevant grid resolution should be about the same as the diameter of the tire. Smaller grid cells will provide detail that is too fine for navigation purposes, while larger cells may obscure the size and shape of relevant obstacles. In this work, Δ=0.4 m. In this case, the maximum curvature that might be measured for the negative obstacle is given by

(16) $$\kappa_{max}=3d/\Delta^{2}$$

The curvature threshold is therefore set to half the maximum value, $\kappa_{0}=\kappa_{max}/2$. From the diagram in Figure 1 it is clear that the minimum measured depth will be at the point defined by $\theta_{fb}$, and this in turn will yield the maximum measured curvature

(17) $$z_{min}\left(r\right)=h-\frac{w-r}{\tan(\theta_{fb})}$$

(18) $$\kappa_{measured}=3z_{min}/\Delta^{2}$$

This places a constraint on the measurement system that can be quantified for a given sensor mount angle and height with Equation (3).

(19) $$z_{min}>\kappa_{0}\Delta^{2}/3$$

where $z_{min}$ is the positive depth.

In addition, when comparing to Equations (13) and (14) to Equation (15), it is clear that there must be at least 5 points covering the hole to perform the stencil calculation. Taking the discretization of the surface into account, there must be a point density of $1/\Delta^{2}$ in the region of the hole. This implies that the threshold for detection with the curvature method is

(20) $$n_{f}+n_{b}>\alpha lw/\Delta^{2}$$

where α is the scale factor representing the required number of points per cell and must be >1. In this work, α=2 is used to ensure adequate point density.

**Figure 4:**
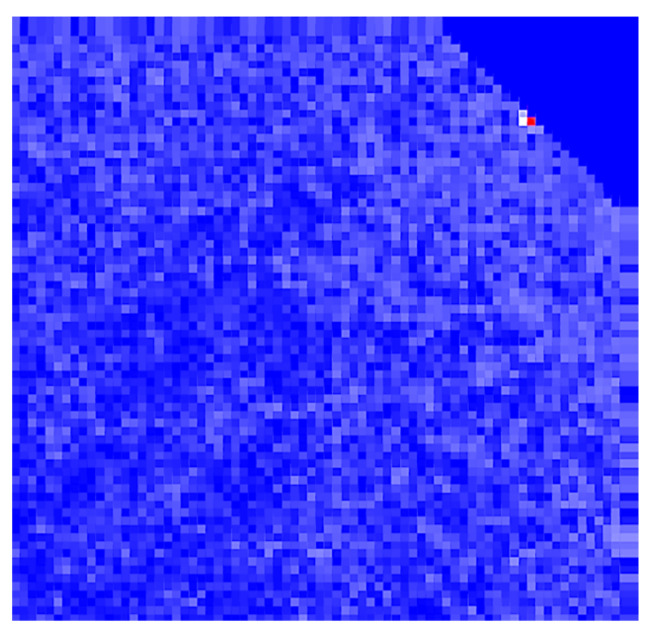
An example surface curvature calculation. Redder regions indicate areas of high curvature. The obstacle region can be seen in the upper right of the image.

Equations (19) and (20) define the requirements for detecting a negative obstacle using the curvature-based method on a grid. In the following sections, it will be shown that the predictions of this analytical model match the results of physics-based simulations of negative obstacle detection with lidar. Figure 4 shows an example of the output of the surface curvature calculation from Equation (15). The curvature was calculated using a VLP-16 sensor mounted at 40 m of height.

## 5. Simulated Experiments

**Figure 5:**
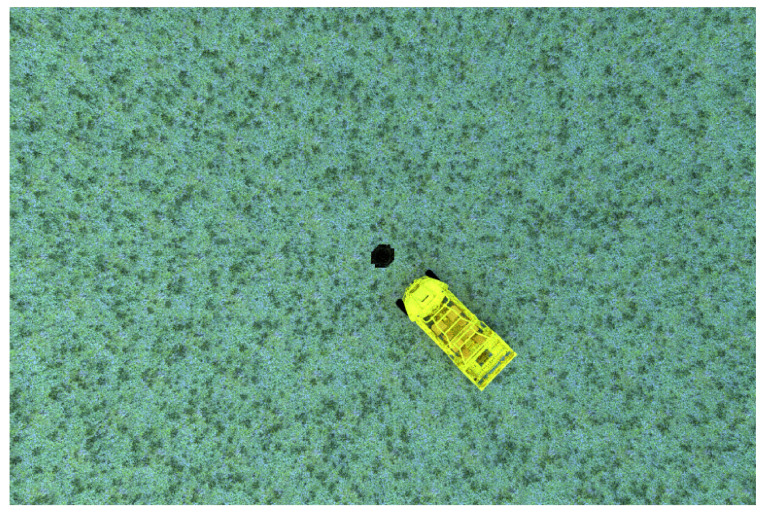
Rendering of the simulated scenario. The UGV moves toward the negative obstacle, a hole which is large enough to damage or immobilize the vehicle.

The simulated scenario is a vehicle navigating through relatively flat terrain toward a negative obstacle. As the vehicle moves toward the negative obstacle, scans are merged into a single point cloud. The combined point cloud is processed at each step, and when the obstacle is detected, the simulation is stopped and the detection range is recorded. The test vehicle is shown near the hole in Figure 5. For the UAV case, the aerial vehicle moves synchronously over the UGV at a prescribed altitude.

The simulated UGV was a Polaris MRZR4 with a roof mounted VLP-16 and simulated RTK localization sensor. The mount height was 2.0 m. For the simulated UAV-mounted lidar, we follow the setup defined in Nakano et al. [9], which had a Velodyne VLP-16 mounted underneath a DJI Matrice 600 Pro. Following the experiments in Nakano et al. [9], the flight altitude was 40 m. The dynamics of the UAV were not simulated in these experiments. Rather, a predefined flight plan was followed.

In this work, the performance parameter is the maximum detection range. In order to optimize the detection range, the lidar sensor should be focused to maximize the look-ahead distance based on the maximum sensor range and the mount height. In this case, the sensor mount angle is given by

(21) $$\gamma =\cos^{-1}h/R$$

Therefore, the mount angle was 88.85${}^{\circ }$ for the UAV-mounted sensor and 68.2${}^{\circ }$ for the UGV-mounted sensor, which has a range of about 100 m.

The most interesting differentiators for negative obstacle detection are holes that are large enough to cause potential damage or immobilization while still being small enough to be difficult to detect. For the purposes of this study, the hole is defined with respect to the size of the MRZR tire, which has a radius of approximately 0.3 m. In this case, a hole of 0.6 m depth and 1.0 m diameter may be large enough to cause immobilization while still being difficult to detect.

For the obstacle detection algorithm, the grid size is set to Δ=0.4 m, ensuring that the negative obstacle, which has a width of 1.0 m, is always covered by at least nine cells. Since the hole has a depth of d=0.6 m, the theoretical curvature threshold (Equation (19)) is $\kappa_{0}=5.625$. Since the hole length and width are both 1.0 m, the point density threshold (Equation (20)) is $(n_{f}+n_{b})>12.5$ total points. It is of note that while this algorithm makes use of surface curvature, the basic requirement of measuring the surface curvature with adequate point density is common to nearly all obstacle detection algorithms. Therefore, the results of this method are relevant to other algorithms that measure surface slope and/or curvature.

### The MSU Autonomous Vehicle Simulator

The predictions of the analytic model are compared to physics-based simulations with MAVS. MAVS uses physics-based lidar models to accurately predict lidar range measurements and account for effects like interaction with vegetation [10] and rain [25]. MAVS has also recently been used to test the performance of obstacle detection algorithms in dense vegetation [26] and to determine the optimal orientation of lidar sensors on an autonomous UGV [27].

In the simulated experiments, a terrain was created with a hole matching the dimensions described above. The hole was not perfectly rectangular but instead had smooth curvature near the sides. The resolution of the surface mesh was 0.125 m. The vehicle and sensor moved directly toward the hole at a constant speed. The initial position of the vehicle and hole were varied by ±0.125 m to ensure than no aliasing affects with the overlay grid occurred. In addition, a small, spatially coherent roughness with a magnitude of 0.05 m was applied to the surface so that it was not perfectly flat. This ensures that, although the analytical model is for idealized conditions, the simulation has more real world variability. Twenty-five trials were conducted at speeds of 2.5–17.5 m/s in 2.5 m/s increments (≈18 m/s being the maximum speed of a DJI Matrice pro), for a total of 175 simulations each for both the UAV and UGV.

## 6. Results

One important way to evaluate negative obstacle detection distance is to compare it to stopping distance for a given speed. The standard formula for stopping distance is [3]

(22) $$R_{stop}=\frac{v^{2}}{2\mu g}+vT_{r}+B$$

where *v* is vehicle velocity, *g* is gravitational acceleration, μ is surface friction, $T_{r}$ is reaction time, and *B* is a safety factor. Following Matthies and Rankin [3], in this work g=9.8, $T_{r}=0.25$, B=2.0, and μ =0.65 for off-road surfaces. If the detection range is less than the stopping distance, then this speed can be considered unsafe for the UGV.

Simulations were performed for three different sensors. Table 3 shows the model parameters for each of these sensors. The UGV-mounted sensors had an elevation of two meters, while, the UAV-mounted sensors had an elevation of 40 m. The tilt angle for each sensor was calculated as a function of the mount height and maximum range using Equation (21).

### 6.1. UGV-Mounted Sensors

**Figure 6:**
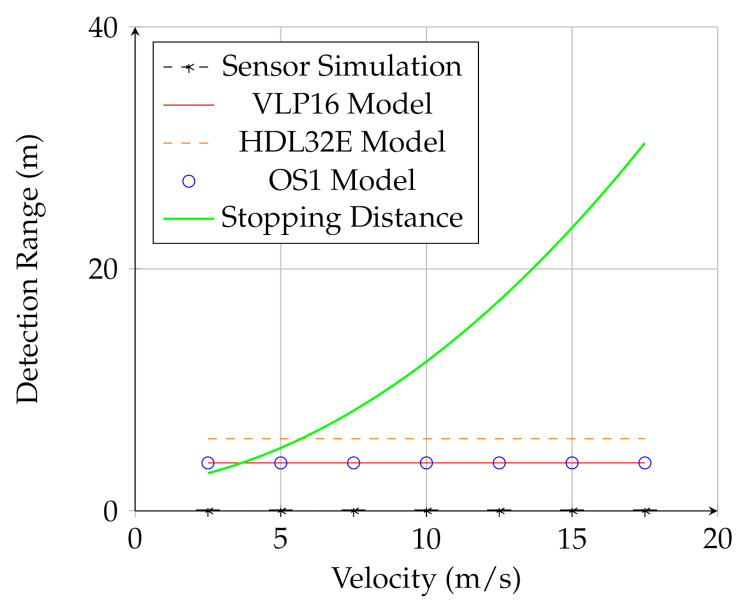
Results for the UGV-mounted sensors. The red line is the analytical model, the black line is the simulation, and the green line is the stopping distance.The dashed black line and marks are the result of the physics-based simulation. The detection range was uniformly 0 for all cases, meaning the algorithm was unable to detect the negative obstacle in any case in the simulation for the UGV-mounted sensor.

For UGV-mounted sensors, the analytical model showed that at the two meter mount height the detection range was quite short. In addition, there was little dependence on speed, indicating that the detection is limited by geometry rather than point density for the UGV-mounted sensor. Figure 6 shows the prediction of the analytical model (the red and orange line and blue circles) as well as the result of the simulation (black line and marks). While the analytical model predicted that UGV-mounted sensor would be able to detect the obstacle at close range, the simulations showed that the UGV-mounted sensor could not detect the negative obstacle at any speed or range. This is because the UGV-mounted lidar did not meet the requirement of Equation (20); mounted at a relatively low elevation, the sensor could not see enough of the bottom of the hole to adequately measure surface curvature in the vicinity of the hole. Therefore, in the case of the UGV-mounted sensor, the analytical model overestimated the ability of the sensor to detect the negative obstacle. One possible explanation for this is the continuous nature of the analytical calculation versus the discrete nature of real lidar scans. The model allows densities to be accumulated as fractions of points, while, in real lidar scanned points are discrete. This difference may be important in these edge cases where the detection range is quite low. Nevertheless, the simulation validates the overall conclusion that the UGV-mounted lidar is not adequate for negative obstacle detection.

### 6.2. UAV-Mounted Sensors

**Figure 7:**
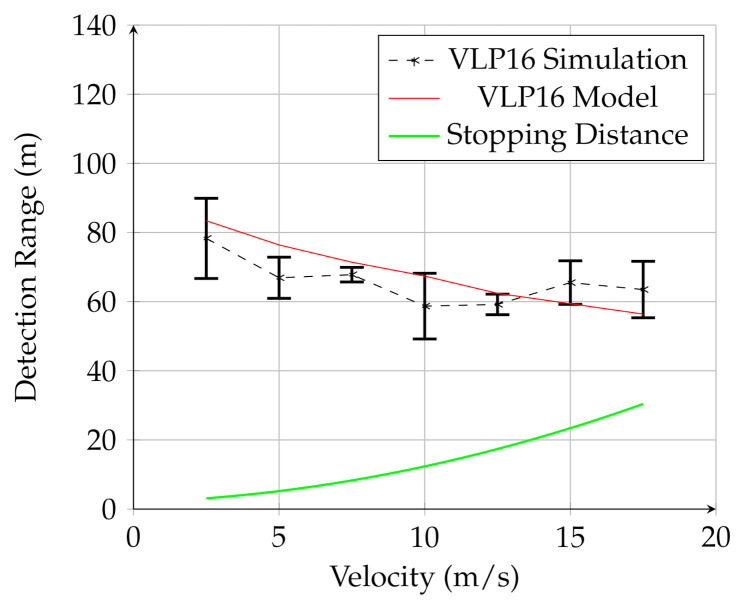
Results for the UAV-mounted VLP-16 sensor. The red line is the analytical model, the black line is the simulation, and the green line is the stopping distance.

**Figure 8:**
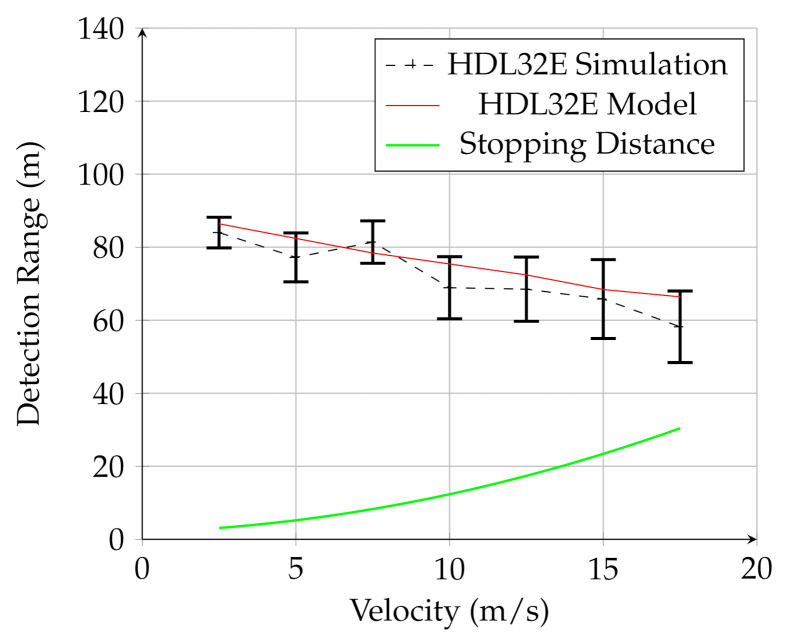
Results for the UAV-mounted HDL-32E sensor. The red line is the analytical model, the black line is the simulation, and the green line is the stopping distance.

**Figure 9:**
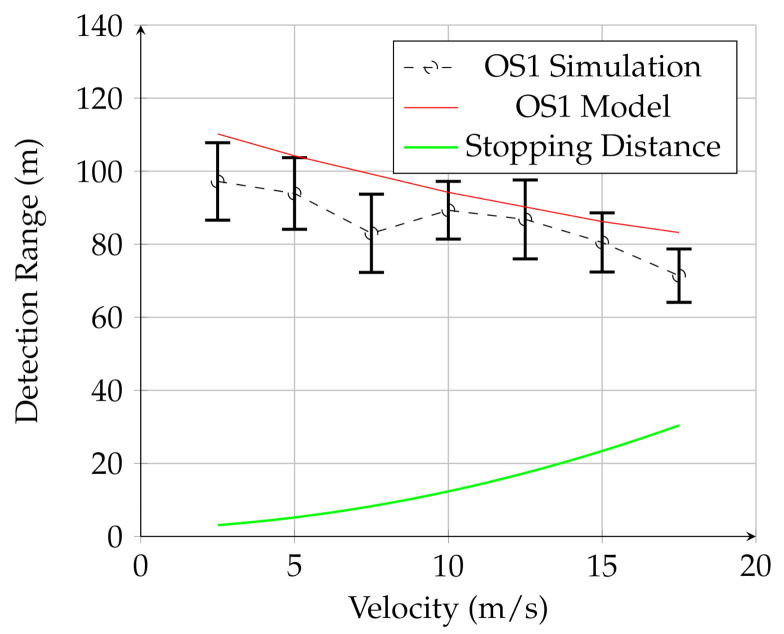
Results for the UAV-mounted OS1 sensor. The red line is the analytical model, the black line is the simulation, and the green line is the stopping distance.

Figure 7, Figure 8 and Figure 9 show the results for the UAV simulation with the VLP-16, HDL-32E, and OS1 sensors, respectively. In these figures, the red line is the prediction of the analytical model, while the dashed black line is the result of multiple physics-based simulations. The error bars are one standard deviation of the distribution of measured results for the 25 trials of the physics-based simulation. The green line is the UGV stopping distance at this speed. The overlap between the red line (model) and black line (simulation) in these figures shows that the analytical model developed in this work provides a reasonable estimate for detection range for a given lidar configuration for UAV mounted lidar. In addition, when comparing to Figure 6, it is clear that the detection range for negative obstacle is greatly increased by using a low-flying UAV. In contrast to the UGV-mounted sensors, the detection range using the UAV-mounted lidar considerably exceeds the stopping distance for all measured velocities.

These figures also demonstrate that the UAV-mounted sensors detect the negative obstacle long before the minimum stopping distance, and that the analytical model and simulation are in reasonable agreement. The results for all three sensors are summarized in Table 4.

Furthermore, of note is the detection probability, $P_{d}$. While the algorithm detected the hole in most of the trials for each sensor, there was some variation between sensors. The HDL-32E had $P_{d}=0.977$, while the OS1 had $P_{d}=0.909$. The VLP-16 had the lowest probability of detection, with $P_{d}=0.886$. There was not significant correlation between $P_{d}$ and speed.

## 7. Discussion

We note two main improvements of the presented analytical model as presented. The first is the improvement over prior models. The second is the capability of the model to distinguish between sensors by taking properties like the sensor resolution, field-of-view, an scan rate into account.

As a point of comparison, consider the analytical model proposed by Matthies and Rankin [3] and Larson and Trivedi [6]. Using the small angle approximation (valid for low sensor altitudes), they propose an equation for estimating the opening angle viewable by the sensor on the back of the negative obstacle (segment E–D in Figure 1). In the notation of this paper, their proposed equation is

(23) $$n_{f(2D)}^{prior}\left(x\right)=\frac{hw}{x(x+w)}$$

**Figure 10:**
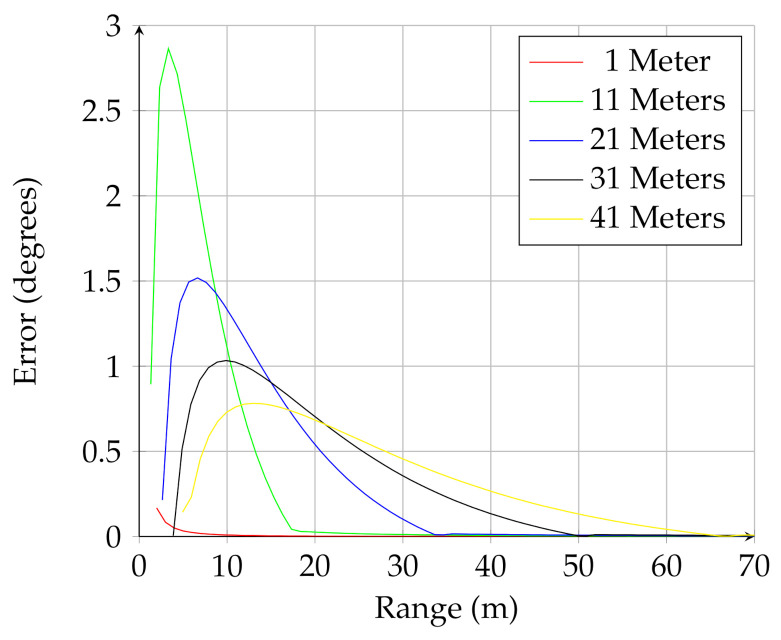
Difference between the analytical model presented in this work and previous models using the small-angle approximation for UGV mounted sensors. The error for different sensor mount heights is shown, with the increase in error shown for sensors at higher elevation angle.

This can be compared to Equation (7) in the current work. While the previous work has the advantage of simplicity, the small-angle assumption is not valid for UAV-mounted lidar. Neglecting the effects of sensor field-of-view, Figure 10 shows how using the full trigonometric calculation presented in this work compare to using the small angle approximation. Figure 10 shows the difference, in degrees, between the two calculations. This figure shows how the small angle approximation overpredicts the viewable angle subtended by the negative obstacle for different sensor elevations.

**Figure 11:**
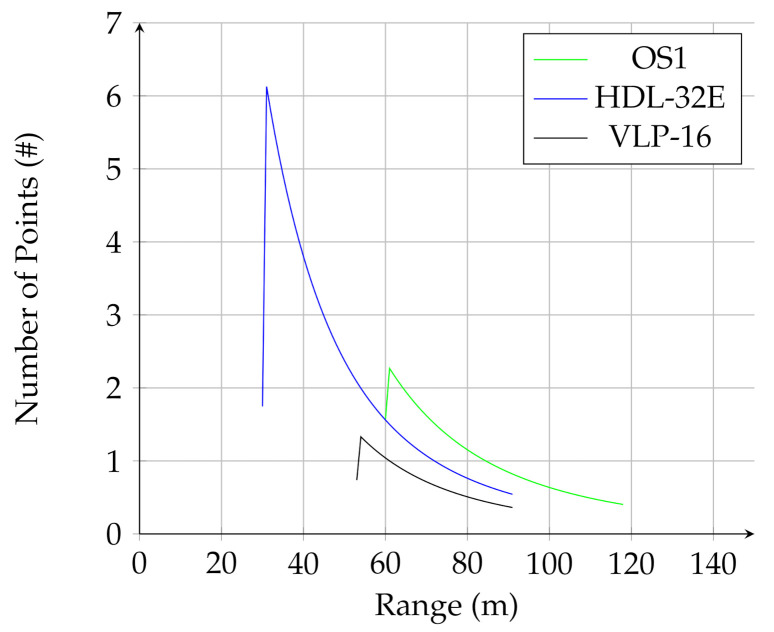
Predicted instantaneous number of points on negative obstacle vs. range for different sensors.

Because the analytical model defined in this work takes different sensor properties into account, it can be used to compare sensors. Figure 11 shows the number of points on the negative obstacle (Equation (10) + Equation (11)) versus the sensor range, for the same scenario discussed in the previous section. The sensor parameters used in the analysis are listed in Table 3.

Figure 11 shows that the OS1 lidar, which has the greatest range, detects the negative obstacle from the farthest distance, while the HDL-32E has the greatest instantaneous number of of points on the obstacle due to the high resolution in the horizontal direction. This demonstrates how the equations presented in the analytical model can be used to optimize sensor selection and deployment for negative obstacle detection.

## 8. Conclusions

An analytical model for predicting the performance of automotive lidar for detecting negative obstacles was developed in this work. The model is valid for all ranges of sensor angles and heights. This model also takes the lidar rotation rate and platform velocity into account. A negative obstacle detection algorithm based on curvature was developed and, coupled to a physics-based simulator, used to cross-validate the analytical model. The analytical model provides a fast, accurate way to estimate the optimal mount height and orientation for a sensor to detect negative obstacles of a specified dimension. The model also provides an estimated safe maximum speed for negative obstacle detection for a given configuration. It was shown that negative obstacle detection ranges for the three UAV-mounted lidar in this study are 60–110 m, depending on the speed of the UAV and the type of lidar used. In contrast, detection ranges for UGV mounted lidar are found to be less than 10 m.

A high-fidelity, physics-based simulator was coupled to a curvature-based obstacle detection algorithm to validate the analytical model. Both the model and simulation show the potential advantages of using a low-flying UAV to detect negative obstacles, providing greater detection range and allowing the UGV to safely operate at higher speeds. The simulation and model agreed well for higher altitudes, but diverged for lower altitudes where the model predicted better detection capabilities than was found with the simulation. Future work in this area will focus on extending the analytical model to include positive obstacles and extended obstacles like vegetation.

## Figures and Tables

**Table 1:** Symbols used in this analysis.

Property	Unit	Symbol
Obstacle Width	meters	*w*
Obstacle Length	meters	*l*
Obstacle Depth	meters	*d*
Sensor Altitude	meters	*h*
Coordinate	meters	*x*
Velocity of Sensor	m/s	*v*
Angle to Far Obs. Edge	rad	$\theta_{ft}$
Angle to Near Obs. Edge	rad	$\theta_{rt}$
Angle to Far Obs. Bottom	rad	$\theta_{fb}$
Angle to Near Obs. Bottom	rad	$\theta_{rb}$
Horiz. Angle Subtended	rad	$\theta_{h}$
Points/Scan, Far Wall	#	$n_{f}^{\prime }\left(x\right)$
Points/Scan, Near Wall	#	$n_{r}^{\prime }\left(x\right)$
Points/Scan, Obs. Bottom	#	$n_{b}^{\prime }\left(x\right)$
Total Points, Far Wall	#	$n_{f}\left(x\right)$
Total Points, Near Wall	#	$n_{r}\left(x\right)$
Total Points, Bottom	#	$n_{b}\left(x\right)$
Total Scans vs. Distance	#	$n_{s}\left(x\right)$
Res. of Surf. Discretization	meters	Δ
Surface Curvature	m${}^{-1}$	κ

**Table 2:** Sensor properties used in this analysis.

Property	Unit	Symbol
Scan Freq	Hz	*f*
Vertical Res	rad	$\delta_{v}$
Horizontal Res	rad	$\delta_{h}$
Max Scan Angle	rad	$\theta_{max}$
Min Scan Angle	rad	$\theta_{min}$
Mount Angle	rad	γ
Sensor Range	m	*R*

**Table 3:** Values for the sensors used in the experiments. All sensors UAV-mounted unless otherwise noted.

Property	VLP16	HDL32E	OS1
Scan Freq (Hz)	10	10	10
Vertical Res (°)	2.0	1.33	0.502
Horizontal Res (°)	0.2	0.17	0.35
Max Scan Angle (°)	15.0	10.6	15.8
Min Scan Angle (°)	−15.0	−30.7	−15.8
Max Range (m)	100.0	100.0	125.0

**Table 4:** Detection ranges measured in physics-based simulations of UAV-mounted lidar with 25 trials at each velocity. The one standard-deviation error bars are listed in parentheses.

Velocity (m/s)	VLP-16 (m)	HDL-32E (m)	OS1 (m)
2.5	78.3 (11.6)	84.0 (4.2)	97.2 (10.6)
5.0	66.9 (6.0)	77.2 (6.7)	93.9 (9.8)
7.5	67.8 (2.1)	81.4 (5.8)	83.0 (10.7)
10.0	58.7 (9.5)	68.9 (8.5)	89.3 (7.9)
12.5	59.2 (3.0)	68.5 (8.8)	86.8 (10.8)
15.0	65.5 (6.3)	65.8 (10.8)	80.5 (8.1)
17.5	63.5 (8.2)	58.2 (9.8)	71.4 (7.3)

## Data Availability

Data is contained within the article.
